# Novel Missense *CNTNAP2* Variant Identified in Two Consanguineous Pakistani Families With Developmental Delay, Epilepsy, Intellectual Disability, and Aggressive Behavior

**DOI:** 10.3389/fneur.2022.918022

**Published:** 2022-07-14

**Authors:** Noor Badshah, Kari A. Mattison, Sohail Ahmad, Pankaj Chopra, H. Richard Johnston, Shakoor Ahmad, Sher Hayat Khan, Muhammad Tahir Sarwar, David J. Cutler, Micheal Taylor, Gayatri Vadlamani, Michael E. Zwick, Andrew Escayg

**Affiliations:** ^1^Institute of Biotechnology and Genetic Engineering, University of Agriculture Peshawar, Peshawar, Pakistan; ^2^Department of Human Genetics, Emory University, Atlanta, GA, United States; ^3^Genetics and Molecular Biology Graduate Program, Graduate Division of Biological and Biomedical Sciences, Laney Graduate School, Emory University, Atlanta, GA, United States; ^4^Department of Animal Health, University of Agriculture Peshawar, Peshawar, Pakistan; ^5^Department of Molecular Biology and Genetics, Institute of Basic Medical Sciences, Khyber Medical University, Peshawar, Pakistan; ^6^Department of Pediatric Neurology, Leeds Teaching Hospital NHS Trust, Leeds, United Kingdom

**Keywords:** epilepsy genetics, *CNTNAP2*, Afridi tribe, Pakistan, autosomal recessive

## Abstract

We report the genetic analysis of two consanguineous pedigrees of Pakistani ancestry in which two siblings in each family exhibited developmental delay, epilepsy, intellectual disability and aggressive behavior. Whole-genome sequencing was performed in Family 1, and we identified ~80,000 variants located in regions of homozygosity. Of these, 615 variants had a minor allele frequency ≤ 0.001, and 21 variants had CADD scores ≥ 15. Four homozygous exonic variants were identified in both affected siblings: *PDZD7* (c.1348_1350delGAG, p.Glu450del), *ALG6* (c.1033G>C, p.Glu345Gln), *RBM20* (c.1587C>G, p.Ser529Arg), and *CNTNAP2* (c.785G>A, p.Gly228Arg). Sanger sequencing revealed co-segregation of the *PDZD7, RBM20*, and *CNTNAP2* variants with disease in Family 1. Pathogenic variants in *PDZD7* and *RBM20* are associated with autosomal recessive non-syndromic hearing loss and autosomal dominant dilated cardiomyopathy, respectively, suggesting that these variants are unlikely likely to contribute to the clinical presentation. Gene panel analysis was performed on the two affected siblings in Family 2, and they were found to also be homozygous for the p.Gly228Arg *CNTNAP2* variant. Together these families provide a LOD score 2.9 toward p.Gly228Arg *CNTNAP2* being a completely penetrant recessive cause of this disease. The clinical presentation of the affected siblings in both families is also consistent with previous reports from individuals with homozygous *CNTNAP2* variants where at least one allele was a nonsense variant, frameshift or small deletion. Our data suggests that homozygous CNTNAP2 missense variants can also contribute to disease, thereby expanding the genetic landscape of *CNTNAP2* dysfunction.

## Introduction

Epilepsy is a neurological disorder characterized by the presence of recurrent and unprovoked seizures, and it is estimated that 70–80% of epilepsy cases have a genetic contribution (1). Discovery of novel genetic variants contributing to epilepsy have largely used experimental designs that favor the discovery of dominant acting alleles (1, 2). As a consequence, while targeted gene panels have yielded a diagnostic rate of ~18% in epilepsy patients, only 4–8% of these cases were due to variants in loci known to cause autosomal recessive (AR) forms of epilepsy (3–5). Whole-exome sequencing (WES) provides a higher diagnostic rate of 20–30% in families with suspected AR epilepsy, illustrating the utility of WES over gene panels when AR inheritance is suspected (6–8). Several genes with broad biological functions have now been associated with AR epilepsy (9). AR forms of epilepsy can be severe with early-onset, such as epilepsy caused by variants in *ALDH7A1*, or have onset in adolescence or later as with variants in *CSTB* (9).

We identified two brothers affected with developmental delay, epilepsy, intellectual disability and aggressive behavior from a consanguineous family (Family 1) belonging to the Afridi tribe within the Federally Administered Tribal Area of the province Khyber Pakhtunkhwa, Pakistan. Consanguineous marriages in this region are common, resulting in a high prevalence of genetically recessive disorders. Through whole-genome sequencing (WGS) we identified 32 genomic regions that were identical by descent between the two brothers. Variant annotation and filtering reduced the candidate regions to six harboring 21 homozygous genetic variants of which four were located within exons of different genes. Segregation of the exonic variants among additional unaffected family members revealed homozygous inheritance of variants in *PDZD7, RBM20*, and *CNTNAP2* in only the affected siblings. A second consanguineous family (Family 2) of Pakistani origin was subsequently identified in which two female siblings exhibited clinical features that were strikingly similar to the affected individuals in Family 1. The two affected members of Family 2 were found to be homozygous for the same *CNTNAP2* variant identified in Family 1.

## Methods

### Participants

#### Family 1

Family 1 is from Pakistan and is of Afridi tribal heritage. Six members from this family participated in this study. Clinical information and blood samples were obtained from all participants after they or a parent/guardian had provided written informed consent.

#### Family 2

Family 2 is also of Pakistani origin. Six members of this family participated in this study. Clinical information and blood samples from the affected siblings and salvia samples from four other family members were obtained after they or a parent/guardian had provided written informed consent.

### Sequencing and Analysis

#### Family 1

Genomic DNA was extracted from whole blood for all participants with the Omega SQ II Blood DNA kit following manufacturer's protocol (D0714, Omega Bio Tek). Paired-end sequencing libraries were generated for individuals V-2 and V-4 using a KAPA HyperPrep kit (Roche, KK8504) following manufacturer protocols. WGS was performed using an Illumina NovaSeq6000 instrument according to manufacturer protocols. Reads were mapped to hg38 using PEMapper and variants (single-nucleotide, SNVs, and indels) called using PECaller (10). Average sequencing depth for both samples was >34X. Runs of homozygosity were identified using the “homozyg group-verbose” function in PLINK (pngu.mgh.harvard.edu/Purcell/plink/) (11).

**Figure 1:**
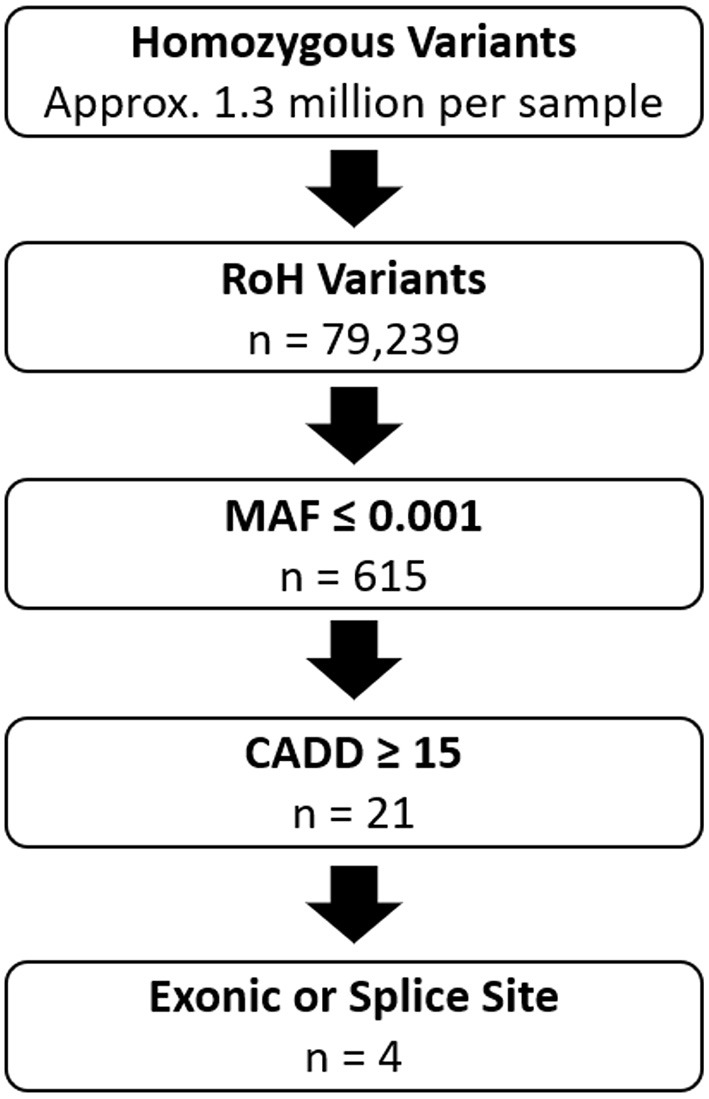
Overview of Variant Filtering in Family 1. RoH Variants: Variants that fall within the 103.8MB of shared homozygous regions between the two affected individuals. MAF ≤ 0.001: Number of variants with minor allele frequency ≤ 0.001 based on data from gnomAD. CADD ≥ 15: Number of variants with CADD scores ≥ 15. Exonic or Splice Site: Number of variants located within exons or splice acceptor or donor sites. n, indicates remaining number of variants after each step.

Variants were annotated using Bystro (bystro.io) (12). Filtering of shared homozygous variants between the affected siblings using the following command: “CADD > 15, MAF <0.001, exonic, splice” (Figure 1) resulted in the identification of exonic variants in *PDZD7, ALG6, RBM20*, and *CNTNAP2*.

#### Family 2

Genomic DNA extracted from whole blood (III-4 and III-5) or from saliva (II-10, II-11, III-2, III-6), Individuals III-4 and III-5 were screened using the National Health Services Genetic Epilepsy Syndromes gene panel (Test ID 59.1) which comprises 402 known and evidence-based epilepsy genes.

### Segregation Analysis

Segregation of variants in *PDZD7, ALG6, RBM20*, and *CNTNAP2* was examined by sequence analysis of all available members of both families. The following primers were used for PCR and sequencing: PDZD7_F-ATGTGTGCCCTTCTCTAACTG andPDZD7_R-GTCCAGGCGAGGGTAAGTT, ALG6_F-TAAGTTGTCTGAGATTCCAGG and ALG6_R-GACAAACAGGCTCCAATC, RBM20_F-AAAGGGAACCGTCTTCTG and RBM20_R-GCATCGCCTCTTTATGTTAAG, CNTNAP2_F-AACAGAGGACTGTCAATTTC and CNTNAP2_R-CAGTCAGAACACACCTAAGT.

### Lollipop Variant Plot

The Lollipop variant plot was generated using freely available source code from https://github.com/pbnjay/lollipops (13). Population variants in gnomAD for *CNTNAP2* were taken from v2.1.1. Human Gene Mutation Database (HGMD) variants for *CNTNAP2* were taken from HGMD Professional version 2021.3. Resulting lollipop diagrams of HGMD and gnomAD variants were merged into a single image. UniProt accession Q9UHC6 was used to draw the protein domains which were overlaid on the final image.

### GSA Analysis

Genomic DNA samples from six members of Family 1 and four members of Family 2 were run on the Illumina Global Screening Array (GSA) v3.0. GSA variants were processed and called using Genome Studio. High-quality, high completeness SNPs called on both the GSA array and by WGS in Family 1 members were merged into a single dataset (302,802 in total) to allow pairwise comparison of Family 1 and Family 2 members. IBD (identity-by-descent) fractions were estimated from observed identity-by-state (IBS) counts.

## Results

### Clinical Presentation and Family History

#### Family 1

**Figure 2:**
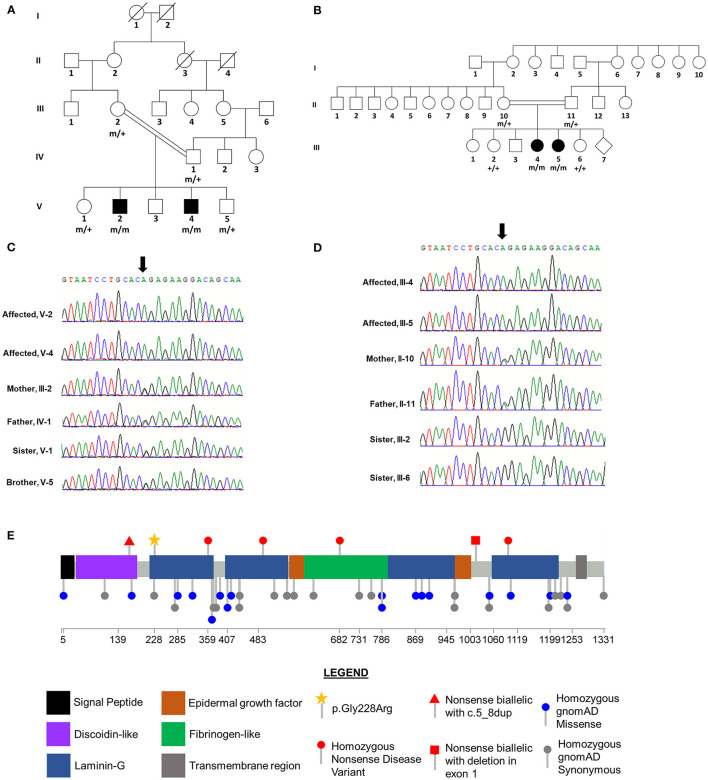
*CNTNAP2* p.Gly228Arg Variant Segregates with Disease in Consanguineous Families. Pedigrees for Family 1 **(A)** and Family 2 **(B)** with affected status (filled symbol) shown. Genotype for *CNTNAP2* variant is shown under individuals who underwent Sanger sequencing. “+” wild-type allele, “m” mutant allele. Sequence traces from Sanger sequencing of *CNTNAP2* in members of Family 1 **(C)** and Family 2 **(D)**. The position of the homozygous c.758G>A variant in the affected siblings is indicated by the arrow. Both parents in each family are heterozygous and unaffected siblings are either heterozygous or wild type for this nucleotide substitution. **(E)** Lollipop plot of previously reported homozygous or biallelic nonsense disease variants in the Human Genetic Mutation Database (HGMD; professional version 2021.3) and homozygous variants in gnomAD (v2.1.1). Star indicates novel homozygous p.Gly228Arg variant identified in this study. Domain and variant locations based on UniProt accession Q9UHC6.

The two affected brothers (V-2 and V-4) are members of a consanguineous family from the Afridi tribe in Pakistan (Figure 2A). Both individuals exhibited normal height, weight and head circumference during the perinatal period. Clinical history of the parents and full siblings of V-2 and V-4 revealed no indication of neurological disease.

Individual V-2 is currently 13 years old. He presented with primary generalized epilepsy onset at 3 years of age. Partial seizure control was achieved with anti-epileptic drugs (AEDs); however, occasional breakthrough seizures still occur (Table 1). Aggressive behavior was observed until 4 years of age. Electroencephalogram (EEG) analysis performed under sedation at 4 years of age identified brief, recurrent, generalized spike and wave discharges.

**Table 1:** Clinical presentation of affected siblings and comparison to previously reported patients.

	**Family 1**		**Family 2**		**Strauss (2006)^a^**	**Gregor (2011)^b^**	**Smogavec (2016)^c^**
	**V-2**	**V-4**	**III-4**	**III-5**			
**Current age**	13 years	10 years	7 years	5 years	NA	NA	NA
**Epilepsy syndrome**	Primary generalized epilepsy	Focal onset epilepsy	Focal onset epilepsy	Focal onset epilepsy	Focal epilepsy	5/7 with epilepsy	Complex focal, generalized epilepsy
**Age of seizure onset**	3 years	2.5 years	3 years	2.5 years	14–20 months	3 months−3 years	5 months−3 years
**Primary seizure type**	Generalized tonic-clonic	Focal onset aware (Partial)	Focal	Left side tonic followed by generalized tonic-clonic	9/9—Focal Onset Aware 4/9—Secondary generalized	Complex	ND
**Current AEDs**	Valproic Acid, Diazepam, Clonazepam, Risperidone	Carbamazepine, Valproic Acid, Levetiracetam, Clonazepam, Methylphenidate	Carbamazepine and Lamotrigine	Carbamazepine, Valproic Acid	ND	ND	Lamotrigine (3/8), Cabamazepine (2/8), Valproate (2/8), Levetiracetam (1/8), Oxcarbazepine (1/8), Phenobarbitone (1/8), Zonisamide (1/8)
**Seizures controlled?**	Some breakthrough seizures	No	No (daily seizures)	No	ND	2/5—No	2/8—No
**Intellectual Disability**	Moderate	Moderate	Yes	Yes	9/9	7/7 (Moderate to Severe)	8/8 (Moderate to Severe)
**Developmental Delay**	Yes, with speech regression	Yes, with speech regression	Yes, poor speech and language	Yes, poor speech abilities	9/9—with speech regression	7/7 4/7–No speech 1/7–Simple Sentences	6/8— <10 words 2/8—Simple sentences
**Autism spectrum disorder**	Yes	Yes	Poor social abilities	Poor social abilities	6/9	ND	ND
**EEG**	4 years: frequent brief generalized high amplitude SWDs	7 years: frequent high amplitude spike discharges in left frontotemporal region	Confirmed seizures of temporal lobe origin	Interictal frontotemporal epileptiform abnormalities and post-ictal temporal slowing on left side	7/9 – normal background, seizures arising from temporal (occasionally frontal) regions with unilateral high-amplitude spike-slow-wave discharges or focal slowing	ND	Generalized slowing (1/8), occasional spikes and slowing left temporal (1/8), frequent left frontotemporal epileptiform discharges (1/8), slow rhythm, sometimes with epileptic discharges (2/8), diffuse cerebral dysfunction (1/8)
**Other Behavioral Features**	Aggression, until 4 years	Aggression	Aggression	Aggression	ND	1/7—Autoagressive behavior	2/8—Aggression 2/8—Notable temper tantrums
**Other Features**	Body movement imbalance (mild)	Body movement imbalance (mild)	Stereotypic hand flapping movements, post-natal microcephaly	Stereotypic hand flapping movements	ND	1/7—Hypotonia	2/8—Hypotonia 2/8—Ataxia

a *Strauss et al. (14)*.

b *Gregor et al. (15)*.

c *Smogavec et al. (16)*.

*AEDs, antiepileptic drugs; NA, not applicable; ND, no data, SWD, spike-wave discharges*.

Individual V-4 is currently 10 years old. He presented with focal-onset epilepsy at 2.5 years of age. AEDs were successful in temporarily decreasing seizure frequency; however, he currently has 1–3 seizures per day. Seizure onset was accompanied by language regression, and he currently speaks few words and is unable to communicate in full sentences. This individual also exhibits severe aggressive behaviors. EEG analysis was conducted under sedation at 7 years of age and recurrent left, fronto-temporal spikes were observed, consistent with a diagnosis of focal epilepsy. Both affected siblings are also diagnosed with intellectual disability and autism spectrum disorder.

#### Family 2

The two affected sisters (III-4 and III-5) are members of a consanguineous family of Pakistani origin (Figure 2B, Table 1). Antenatal, birth and early development during the first year were normal. Routine blood tests, and serum, urine and cerebrospinal fluid metabolic analyses were normal. No structural brain abnormalities were visible via MRI in either sibling. Clinical history of the parents and full siblings of III-4 and III-5 revealed no indication of neurological disease.

III-4 is currently 7 years old. She presented with focal onset seizures at 3 years of age. She experiences hypermotor movement of limbs, and eye deviation to the right during her seizures which typically last 1 min. She currently has daily seizures despite AED treatment. EEG analysis confirmed epileptic seizures of temporal lobe origin.

III-5 is currently 5 years old. She presented with an explosive onset of seizures at 2.5 years of age. She experiences pallor, eye deviation to the left, tonic seizures affecting the left side followed by generalized tonic-clonic seizures. Her seizures last from 2 to 5 min and typically occur in clusters. Partial seizure control was obtained through AED treatment. EEG analysis showed interictal frontotemporal epileptiform abnormalities and post ictal temporal slowing on the left side, but seizures have not been captured. Both affected sisters exhibited significant learning difficulties, poor social abilities, and aggressive behaviors by 4 years of age.

### Genomic Analyses

#### Family 1

The affected siblings had not previously undergone genetic testing. Given the consanguineous nature of the family, we hypothesized that a homozygous variant shared by the two affected siblings was the most likely explanation for their clinical features. We performed WGS on genomic DNA isolated from whole-blood samples from the two affected brothers.

Homozygosity analysis revealed that 32 genomic regions, totaling 103.8 MB, were shared by the two affected brothers and homozygous variants within these regions were identified. Variants, either SNVs or indels with CADD scores ≥ 15 and minor allele frequency (MAF) ≤ 0.001 were selected for further analysis (Figure 1, Supplementary Table 1). After filtering for variants in exons or splice sites, four candidate variants were identified: *PDZD7* (c.1348_1350delGAG, p.Glu450del), *ALG6* (c.1033G>C, p.Glu345Gln), *RBM20* (c.1587C>G, p.Ser529Arg), and *CNTNAP2* (c.785G>A, p.Gly228Arg) (Table 2).

**Table 2:** Segregation of candidate variants.

**Gene**		***CNTNAP2***	***ALG6***	***RBM20***	***PDZD7***
**Chromosome**		7	1	10	10
**HGVS DNA**		NM_014141.6:	NM_013339.4:	NM_001134363.3:	NM_001195263.2:
		c.682G>A	c.1033G>C	c.1587C>G	c.1348_1350delGAG
**HGVS Protein**		p.Gly228Arg	p.Glu345Gln	p.Ser529Arg	p.Glu450del
**CADD Score**		21.4	28.2	32	23.3
**Population Counts^a^**	**gnomAD**	2:0	15:0	12:0	237:3
	**GMEVariome^b^**	0:0	1:0	0:0	0:0
	**GenomeAsia 100K^c^**	0:0	0:0	0:0	0:0
**Family 1**	**V:2**	Hom	Hom	Hom	Hom
	**V:2**	Hom	Hom	Hom	Hom
	**III:2**	Het	Het	Het	Het
	**IV:1**	Het	Het	Het	Het
	**V:1**	Het	Hom	Het	Het
	**V:5**	Het	Het	Het	Het
**Family 2**	**III:4**	Hom	WT	WT	WT
	**III:5**	Hom	WT	WT	WT
	**II:10**	Het	WT	WT	WT
	**II:11**	Het	WT	WT	WT
	**III:2**	WT	WT	WT	WT
	**III:6**	WT	WT	WT	WT

a *Population counts indicate number of times allele was observed. Shown as Het:Hom*.

b *GMEVariome is a collection of sequence data from individuals in the Greater Middle East, including Pakistan (17)*.

c *GenomeAsia 100K is a database of Asian individuals, including South Asians (18)*.

*Het, heterozygous; Hom, homozygous*.

Sanger sequencing was used to determine the segregation of the four candidate variants in the parents (III-2 and IV-1), an unaffected brother (V-5), and an unaffected sister (V-1). The variants in *PDZD7, RMB20*, and *CNTNAP2* co-segregated with disease status, occurring in a homozygous state in the two affected brothers and a heterozygous state in the four other family members (Figure 2C, Table 2). The *ALG6* variant was homozygous in an unaffected sibling (V-1), indicating that it was unlikely to be pathogenic.

#### Family 2

Gene panel testing in the two affected sisters (III-4 and III-5) revealed that both siblings were homozygous for the same *CNTNAP2* c.682G>A (p.Gly228Arg) variant that was first found in the affected brothers in Family 1. Sanger sequencing for this variant in the other available family members confirmed co-segregation with disease status, with it only being detected in the homozygous state in the affected siblings. As expected, each parent (II-10 and II-11) was heterozygous for this variant, and it was absent in the two unaffected siblings (III-2 and III-6, Figure 2D). The variants detected in *PDZD7, RMB20*, and *ALG6* in Family 1 were not observed in Family 2 (Table 2).

### Relatedness and Linkage Analysis

Given the rarity of this variant in other populations, and that it was observed twice in the same relatively isolated population, both times as a homozygote only found in affected individuals from a consanguineous family, it seems likely that the *CNTNAP2* p.Gly228Arg variant has been inherited by both Family 1 and 2 from some common ancestor, rather than being the result of two different *de novo* mutational events. Nevertheless, we know Family 1 and 2 are two independent observations of the association between genotype and phenotype as the affected individuals are different sexes and ages and cannot be the result of the same probands being discovered by two different investigators.

However, given the likelihood that *CNTNAP2* p.Gly228Arg is a shared mutation from a common ancestor, it is important to understand the extent to which the rest of the genome is also shared. IBD analysis via PLINK suggests that no more than 1/32 of the genome is shared IBD between any member of Family 1 and Family 2, with insufficient power to rule out less of the genome being shared. Thus, while these families could be as close as 2–3rd cousins, there is no evidence that any individual in Family 1 is closely related to any individual in Family 2. As a positive control, within families all members had estimated relatedness consistent with known relatedness.

To further examine the structure of the disease haplotype, we compared the SNP content of the 140 kb interval encompassing the *CNTNAP2* p.Gly228Arg variant in both families. While this region was homozygous in affected individuals within both families, differences in the SNP content of the disease haplotype were observed between the families. However, a conserved interval of ~20 kb spanning 5 SNPs flanking the disease variant was identified between the two families. These observations are consistent with the likelihood of these families being distantly related. Thus, since overall genomic sharing is quite low between Family 1 and Family 2, we should view the fact two families show the same phenotype-genotype pattern at *CNTNAP2* p.Gly228Arg as fundamentally independent replications.

Although these families are small and only two in number, we can formally perform a linkage analysis. Under the hypothesis that *CNTNAP2* p.Gly228Arg is the only recessive, 100% penetrant (no recombination distance) variant contributing to disease in these families, the likelihood of the observation under this hypothesis in each family is 1.0, and the likelihood of the data under chance segregation for each family is: $\frac{1}{4}*\frac{1}{4}*\frac{3}{4}*\frac{3}{4}=\frac{9}{256}=0.03156$. Thus, both families provide a LOD score of ~1.45 with a combined LOD of 2.9. This is the maximum possible LOD score for a recessive hypothesis with 2 affected and 2 unaffected sibs in each family.

## Discussion

We first performed genetic analysis on two brothers from a consanguineous family of Afridi tribal heritage who presented with developmental delay, epilepsy, intellectual disability, and aggressive behavior. Analysis of 32 regions of homozygosity shared by the affected siblings revealed four candidate variants: *PDZD7* (p.Glu450del), *ALG6* (p.Glu345Gln), *RBM20* (p.Ser529Arg), and *CNTNAP2* (p.Gly228Arg).

*PDZD7* encodes a scaffolding protein expressed in the cilia of inner ear hair cells and photoreceptors (19, 20). Pathogenic *PDZD7* variants have been identified in individuals with AR non-syndromic hearing loss and it has been suggested that *PDZD7* variants may also modify the severity of Usher syndrome (19, 21, 22). The lack of overlap between the clinical phenotypes associated with *PDZD7* mutations and the affected siblings suggests that the p.Glu450del variant is unlikely to be pathogenic. Furthermore, this variant is seen three times in a homozygous state in gnomAD which excludes individuals with severe pediatric-onset illnesses (Table 2).

*ALG6* encodes the enzyme Man_9_GlcNAc_2_-P-P-Dol α-1,3-glucosyltransferase. Patients with pathogenic *ALG6* variants have abnormal accumulation of Man_9_GlcNAc_2_-P-P-Dol and hypoglycosylation of proteins which contribute to autosomal recessive congenital disorder of glycosylation (CDG) Type Ic (or ALG6-CDG) (23, 24). ALG6-CDG is typically characterized by developmental delay, seizures and speech disabilities (25). However, an unaffected sibling (V-1) was also homozygous for the *ALG6* p.Glu345Gln variant, indicating that it is unlikely to be pathogenic.

*RMB20* encodes the RNA-binding motif protein #20 which is highly expressed in striated muscle and functions as a splicing factor (26, 27). Pathogenic variants in *RBM20* have been identified in patients with autosomal dominant (AD) dilated cardiomyopathy (28, 29). The identified p.Ser529Arg variant falls within the RNA recognition motif (RRM) which plays an important role in splicing regulation, and mutations within the RRM have been previously identified in individuals with AD dilated cardiomyopathy (30–33). The cardiac phenotypes associated with RBM20 variants are inconsistent with the neurological features observed in the affected siblings. However, since this gene has been shown to regulate splicing of some neuronally expressed transcripts, such as *CACNA1C* in which epilepsy mutations have been previously reported, we cannot exclude the possibility that the p.Ser529Arg variant might contribute to the clinical features in the affected siblings from Family 1 (27, 34).

*CNTNAP2* encodes the contactin-associated protein-like 2 which is a transmembrane protein that facilitates axo-glial contacts in mature myelinated axons and is expressed throughout the brain with highest expression in the cortex, hippocampus, substantia nigra, interpeduncle nucleus, pontine nucleus and medial mammillary nucleus (35, 36). The homozygous *CNTNAP2* frameshift variant, c.3709delG, identified in a consanguineous Old-Order Amish family, was the first reported mutation for cortical dysplasia-focal epilepsy syndrome (14). Zweier and colleagues identified the homozygous splice acceptor variant, c.1671-1G>T, in a consanguineous family with Pakistani ancestry exhibiting Pitt-Hopkins-like syndrome (37). Additionally, homozygous deletions of various sizes within *CNTNAP2* have also been identified in patients with epilepsy, intellectual disability and/or autism spectrum disorder (15, 16, 38–40). Segregation analysis in Family 1 demonstrated that the c.785G>A (p.Gly228Arg) missense variant, located in the first laminin-G domain, only occurred in a homozygous state in the affected siblings, consistent with a recessive mode of inheritance (Figures 2C,E, Table 2). Furthermore, this variant was not observed in the homozygous state in gnomAD, GMEVariome, or GenomeAsia100K, and was only observed twice in the heterozygous state in gnomAD (Table 2). Approximately 20% of individuals in gnomAD are of Asian descent, GMEVariome contains 2,497 unrelated individuals of Middle Eastern ancestry, and GenomeAsia100K comprises 1,739 unrelated individuals with 113 from Pakistan (17, 18).

During the course of this study, we became aware of a second consanguineous family of Pakistani origin (Family 2) in which the two affected siblings were found to be also homozygous for the c.785G>A (p.Gly228Arg) variant in *CNTNAP2*. Clinical presentation in the two affected sisters in Family 2 showed striking overlap with the affected siblings from Family 1, with all four affected individuals exhibiting developmental delay, epilepsy, intellectual disability, and aggressive behaviors (Table 1). Segregation of the *CNTNAP2* variant within Family 2 confirmed co-segregation with disease status (Figures 2B,D). The additional candidate variants observed in Family 1 were not detected in Family 2, providing additional support for the pathogenicity of the *CNTNAP2* variant (Table 2). Together these families give a LOD of 2.9 in support of c.785G>A being a recessive, fully penetrant disease causing variant.

Prior to the current study, all reported disease-associated *CNTNAP2* variants were homozygous or biallelic deletions, splice site variants or nonsense variants which would be predicted to abolish protein function (14, 16, 37, 40). Mice lacking *Cntnap2* (*Cntnap*2^−/−^) recapitulate the seizure and core autism-related behavioral phenotypes seen in patients and reveal a number of pathophysiological alterations including neuronal migration abnormalities, asynchronous neuron firing patterns, and a reduced number of GABAergic interneurons (41). However, the mechanism by which missense variants result in disease is less clear. Recent work in *in vitro* cell culture models has shown that loss of one *CNTNAP2* allele is sufficient to impact axonal growth and heterozygous missense variants identified in patients with autism were associated with deficits in protein trafficking (36). No neurodevelopmental or neuropsychiatric abnormalities were noted in heterozygous carriers of the *CNTNAP2* p.Gly228Arg variant from either family.

In addition to epilepsy and intellectual disability, there are other striking similarities between other clinical features of the affected siblings in the current families and previous reports (Table 1). For example, Smogavec and colleagues described eight patients with homozygous *CNTNAP2* variants of which six patients were non-verbal or had very limited speech ability (16). Additionally, two patients exhibited aggressive behaviors and three were reported to have notable temper tantrums (16).

Taken together, the genetic analysis of the two families and clinical similarities between the affected siblings and other patients with *CNTNAP2* variants strongly support that the identified p.Gly228Arg variant is likely causative. Functional analysis of this variant will be required to establish the impact on CNTNAP2 function and the underlying disease mechanism. This is the first demonstration of co-segregation of a homozygous *CNTNAP2* missense variant within an affected pedigree. Further work to functionally evaluate both homozygous and heterozygous missense *CNTNAP2* variants will aid in the identification of genotype-phenotype correlations and may contribute to the development of more efficacious treatments for these patients.

## Data Availability Statement

The datasets presented in this study can be found in online repositories. The names of the repository/repositories and accession number(s) can be found below: National Center for Biotechnology Information (NCBI) BioProject, https://www.ncbi.nlm.nih.gov/bioproject/, PRJNA695560 and NCBI ClinVar, https://www.ncbi.nlm.nih.gov/clinvar/, SCV001478474.

## Ethics Statement

The studies involving human participants were reviewed and approved by Ethical Committee at the Institute of Biotechnology and Genetic Engineering, the University of Agriculture, Peshawar, Pakistan. Written informed consent to participate in this study was provided by the participants' legal guardian/next of kin. Written informed consent was obtained from the individual(s), and minor(s)' legal guardian/next of kin, for the publication of any potentially identifiable images or data included in this article.

## Author Contributions

NB and KM wrote the manuscript. NB collected clinical information and blood samples. KM identified variants, analyzed variant predicted pathogenicity, and performed segregation analysis. PC performed ROH analysis. HJ performed quality control, mapping, and calling of WGS data. DC performed GSA/Relatedness analysis. MT and GV collected clinical information, blood and saliva samples, and assisted with editing of the manuscript. SoA, ShA, SK, and MS assisted with experimental design. MZ and AE contributed to the experimental design and the writing of the manuscript. All authors contributed to the article and approved the submitted version.

## Funding

This work was partially supported by an International Research Support Initiative Program award from the Higher Education Commission of Pakistan to NB. KM was supported by a training grant appointment (5T32GM008490). Additional support was provided by the Emory University Research Council (AE and MZ) and the Georgia Clinical & Translational Science Alliance of the National Institutes of Health [UL1TR002378].

## Conflict of Interest

The authors declare that the research was conducted in the absence of any commercial or financial relationships that could be construed as a potential conflict of interest.

## Publisher's Note

All claims expressed in this article are solely those of the authors and do not necessarily represent those of their affiliated organizations, or those of the publisher, the editors and the reviewers. Any product that may be evaluated in this article, or claim that may be made by its manufacturer, is not guaranteed or endorsed by the publisher.
