# Supplementary material for: Novel Missense CNTNAP2 Variant Identified in Two Consanguineous Pakistani Families With Developmental Delay, Epilepsy, Intellectual Disability, and Aggressive Behavior
Source: Front Neurol. 2022 Jul 14;13:918022. doi: 10.3389/fneur.2022.918022 (PMC9329621; doi:10.3389/fneur.2022.918022)
Supplement: Supplementary file 1 [file Table_1.DOCX]

Supplementary Material

**Supplemental Table 1.** **Shared Variants Identified in the Affected Brothers.**

Homozygous variants with MAF ≤ 0.001, CADD ≥ 15.

^a^Genomic position is based on hg38.

^b^gnomAD frequency represents homozygous allele frequency.

Abbreviations: Alt, alternate allele; CADD, Combined Annotation Dependent Depletion; Chr, chromosome; DEL, Deletion; Ref, reference allele; SNV, single nucleotide variant

| **Chr** | **Position^a^** | **Ref** | **Alt** | **Variant Type** | **Location** | **Gene** | **dbSNP ID** | **CADD Score** | **gnomAD frequency^b^** |
| --- | --- | --- | --- | --- | --- | --- | --- | --- | --- |
|  |  |  |  |  |  |  |  |  |  |
| 1 | 63419415 | G | C | SNV | exonic | ALG6 | rs142707911 | 28.2 | 0 |
| 1 | 69324851 | C | T | SNV | intergenic |  | rs559114975 | 19 | 0 |
| 3 | 174840870 | T | A | SNV | intergenic |  | rs545360340 | 15.4 | 0.000031859 |
| 3 | 176594852 | C | T | SNV | intergenic |  | rs369457267 | 21.8 | 0.000031849 |
| 4 | 13371261 | C | A | SNV | intronic |  | rs937001005 | 15.5 | 0 |
| 7 | 147108278 | G | A | SNV | exonic | CNTNAP2 | rs371512835 | 21.4 | 0 |
| 10 | 101018271 | CTC | - | DEL | exonic | PDZD7 | rs555444131 | 23.3 | 0.000010614 |
| 10 | 101249392 | G | A | SNV | intergenic |  | rs548280240 | 22 | 0 |
| 10 | 101901487 | T | G | SNV | intronic |  | rs569668688 | 15.2 | 0 |
| 10 | 110797567 | C | G | SNV | exonic | RBM20 | rs539932441 | 32 | 0 |
| 10 | 110851117 | T | A | SNV | intergenic |  | rs372591825 | 19.3 | 0 |
| 10 | 110858145 | G | A | SNV | intergenic |  | rs368141988 | 16.2 | 0 |
| 10 | 110921574 | T | G | SNV | intronic |  | rs187395797 | 16.3 | 0 |
| 10 | 110985777 | G | A | SNV | intronic |  | rs201258692 | 17.3 | 0.000548420 |
| 10 | 111004594 | T | - | DEL | intronic |  | rs730881016 | 16.1 | 0.000556479 |
| 10 | 111010957 | C | T | SNV | intronic |  | rs370486167 | 16.3 | 0 |
| 21 | 24018297 | G | A | SNV | intergenic |  | rs189938410 | 22.5 | 0 |
| 21 | 25549608 | T | A | SNV | intergenic |  | rs551677808 | 17.9 | 0 |
| 21 | 31174041 | G | A | SNV | intronic |  | rs184146552 | 16 | 0 |
| 21 | 32678632 | A | T | SNV | intronic |  | rs75688000 | 15.7 | 0.000009427 |
| 21 | 36067727 | T | G | SNV | intergenic |  | rs914317800 | 15.1 | 0 |
